# Anti-Fatigue Effects of Yogurt Fermented with *Lactobacillus delbrueckii* subsp. *bulgaricus* OLL1073R-1 in Healthy People Suffering from Summer Heat Fatigue: A Randomized, Double-Blind, Placebo-Controlled Trial

**DOI:** 10.3390/nu10070798

**Published:** 2018-06-21

**Authors:** Seiya Makino, Jun Hemmi, Hiroshi Kano, Mari Kashiwagi, Kenichi Hojo, Yukio Asami

**Affiliations:** 1 Food Microbiology Research Laboratories, R&D Division, Meiji Co., Ltd., 1-29-1 Nanakuni, Hachiouji, Tokyo 192-0919, Japan; jun.henmi@meiji.com (J.H.); hiroshi.kano@meiji.com (H.K.); yukio.asami@meiji.com (Y.A.); 2 Food Development Laboratories, R&D Division, Meiji Co., Ltd, 1-29-1 Nanakuni, Hachiouji, Tokyo 192-0919, Japan; mari.kashiwagi@meiji.com (M.K.); kenichi.houjou@meiji.com (K.H.)

**Keywords:** anti-fatigue, yogurt, summer heat, stress

## Abstract

Fatigue caused by summer heat is a typical indefinite complaint along with anorexia, loss of sleep, stress, lack of motivation and, in some cases, catching a cold. Yogurt fermented with *Lactobacillus delbrueckii* subsp. *bulgaricus* OLL1073R-1 has been shown to stimulate the immune system and reduce the risk of catching colds. Here, we conducted a randomized, double-blinded, placebo-controlled trial to investigate whether ingesting this yogurt could ameliorate summer heat fatigue in 49 healthy males (median age 40.0 ± 6.0 years; 30–49 years) who felt fatigued every summer. Fatigue was evaluated by visual analogue scales (VAS) and the balance of sympathetic/parasympathetic nervous systems. After 12 weeks of ingestion in early autumn, the VAS fatigue scores in the yogurt group were lower than those of the placebo group. These results indicate that yogurt fermented with *L. bulgaricus* OLL1073R-1 can ameliorate summer heat fatigue lasting up to early autumn.

## 1. Introduction

Global warming is a world-wide problem that affects the health and productivity of working individuals [1]. High temperatures in the summer increase the incidence of death by heatstroke and are also expected to increase summer heat fatigue, recognized as symptoms resulting mainly from the dysfunction of the autonomic nervous system, such as chronic fatigue, anorexia, loss of sleep, stress, lack of motivation and so on. Currently, many people might suffer from summer heat fatigue, but there are no established treatments or therapeutic agents as it is not recognized as an illness because of its broad symptoms. In summer heat fatigue, dysfunction of the immune system is expected to occur similar to that in patients with chronic fatigue syndrome [2] but it still remains unclear.

Potent anti-fatigue activities of foods, plants, medical herbs, and lactic acid bacteria have been reported previously [3,4,5,6,7,8]. Some of the active components were expected to be polysaccharides [9,10], and polysaccharides purified from fungi, marine algae, and green tea were shown to exhibit anti-fatigue effects in animal studies after physiological challenges such as swimming and running [11,12,13]. However, few human trials have investigated the effects of food or its components on the chronic fatigue that healthy people suffer in daily life as in the case of summer heat fatigue.

Our previous study showed that *Lactobacillus delbrueckii* subsp. *bulgaricus* OLL1073R-1 produces high amounts of exopolysaccharide (EPS), which can induce interferon (IFN)-gamma production and augment natural killer (NK) cell activity in mice [14,15]. In the elderly, ingestion of yogurt fermented using this strain was also shown to augment NK cell activity and reduce the risk of catching colds [16]. Improved quality of life (QOL) in areas such as “Lacks general motivation”, “Irritation”, “Stress,” and “Easily fatigued” were also observed in this study [16].

In the present study, we evaluated the effects of yogurt fermented with *L. bulgaricus* OLL1073R-1, which contains immunostimulatory EPS, on fatigue in the period between the summertime and early autumn. Scores of the visual analogue scale (VAS) related to fatigue were lower in the yogurt group compared to the placebo group during the latter half of the ingestion period. To our knowledge, this is the first report demonstrating the anti-fatigue effects of yogurt in a human trial.

## 2. Materials and Methods

### 2.1. Study Design

This study was designed as a randomized, double-blind, placebo-controlled study conducted at the medical corporation bokushinkai CLINTEXE clinic, Tokyo, Japan between 26 June 2015 and 26 September 2015. The reporting of this trial follows the recommendations of the CONSORT (Consolidated Standards of Reporting Trials) 2010 statement [17]. This trial was registered at the University Medical Information Network Clinical Trial registry (UMIN-RCT Identifier UMIN000025532).

### 2.2. Participants

We enrolled 106 males who felt summer heat fatigue every summer because their yogurt intake was less frequent than that of females. Inclusion criteria were residents of Tokyo and its suburbs, aged between 30 and 49 years, body mass index (BMI) between 18.5 and 29.9, non-smokers, day-shift desk workers, with stable dietary habits. Exclusion criteria included presence of immunodeficiency, patients with malignancy, outpatients or patients requiring drug treatment, allergies to food or medicines, lactose intolerance, regular intake of alcohol at more than 60 g/day, more than 2 intakes per week of fermented milk or beverages containing lactic acid bacteria in the past 3 months, intake habits for antibiotics, laxatives, functional foods, or supplements containing oligosaccharides, dietary fibers, or lactic acid bacteria in the past 3 months, participants of other clinical studies in the past 1 month. After assessment for eligibility, 50 participants with NK cell activities near the median were selected. The sample size was determined with reference to a previous double-blind, placebo-controlled, cross-over trial with 20 volunteering participants [18]. All subjects gave written informed consent, and the study was approved by the ethics committee at the medical corporation bokushinkai CLINTEXE clinic and Meiji Co., Ltd. The ethical approval codes were 2015-0527-01 and 52, respectively. The study was designed to comply with the Declaration of Helsinki.

### 2.3. Intervention

All participants were randomized to receive either yogurt fermented with *L. bulgaricus* OLL1073R-1 and *Streptococcus thermophilus* OLS3059 (yogurt) or a placebo and ingested a bottle of 100 mL per day for 12 weeks. These strains were originally isolated from Bulgarian traditional yogurt. Final acidity of the yogurts was 0.76 to 0.90% and it contained EPS ≥ 2.9 mg/100 mL, as measured by the phenol-sulfuric acid method [16]. The placebo was acidified milk prepared by adding lactic acid to the same acidity as that in yogurt. The both test foods were prepared in the same plastic bottles by the R&D division in Meiji Co., Ltd. The ingredients were milk, skimmed milk, high-fructose corn syrup, sugar and food additives, such as pectin. Table 1 shows the composition of each food.

Randomization was performed using a computer-generated allocation sequence with allocation factors, age, NK cell activity, and VAS (“general malaise”, “feeling languid” and “fatigue”).

### 2.4. Schedule and Temperature during the Study

**Figure 1:**
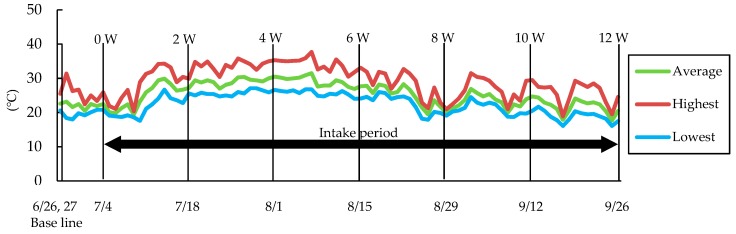
Temperatures in Tokyo during the study. Data were obtained from the meteorological agency in Japan.

Participants visited the clinic for measurements at 6 or 7 days before the first intake day (baseline) and at every 2 weeks from the first intake day to the end of the 12-week intake period (2 W, 4 W, 6 W, 8 W, 10 W, and 12 W). The temperature at Tokyo during this study is shown in Figure 1. The beginning of an increase in the average temperature in summer was observed around the 6th day after first intake, on 10th July 2015, and the high temperature was maintained from 2 W to 6 W. After 6 W, the temperature decreased and increased repeatedly up to 12 W.

### 2.5. Primary Outcome Measures

The primary outcome measures were the between-group differences in the scores of questionnaires associated with the potent symptoms of summer fatigue (VAS, Profile of Mood States (POMS), Face Scale) and NK cell activities.

### 2.6. Secondary Outcome Measures

The secondary outcome measures were the between-group differences in the autonomic nervous system (balances of the sympathetic/parasympathetic nervous systems), immune parameters (cumulative incidence and duration of catching colds).

### 2.7. Measurements

Questionnaires (VAS, POMS, Face Scale), blood sampling, physical examination, and measurements for the nervous system were conducted at 6 or 7 days before the first intake (baseline) and at every 2 weeks from the day of first intake to the end of the 12-week intake period (2 W, 4 W, 6 W, 8 W, 10 W, 12 W). The summer fatigue symptom questionnaires contained the following 13 items: general malaise, feeling languid, fatigue, lack of appetite, difficulty falling asleep, sleep quality, daytime sleepiness, psychological stress, irritation, motivation, thinking power, thirst, and exhilaration. Feelings were evaluated using VAS, on a scale of 0 (no symptoms) to 10 (very severe symptoms) according to the guideline of the Japanese Society of Fatigue Science. In addition, subjects answered the degree of summer heat fatigue during the intake period by the following grades: none, slight, moderate, severe. Blood samples were collected in the morning after an overnight fast. NK cell activities were measured by the ^51^Cr release method at the Biotherapy Institute of Japan, Inc. (Tokyo, Japan). The balance of the sympathetic/parasympathetic nervous systems was evaluated by using an autonomic nervous system sensor VM302 (FMCC Co., Ltd., Osaka, Japan). 

All participants were instructed to track any symptoms they felt on a “cold symptom checklist” if they noted symptoms for the common cold. The common cold symptoms in this checklist included the following: cough, sneezing, stuffy nose, runny nose, sore throat, red throat, swollen throat, head ache, temperature, ache, painful articulation, fatigue, and loss of appetite [16]. Finally, using this checklist and the participants’ body temperature records, a medical doctor confirmed whether each subject had contracted a common cold during the study.

### 2.8. Statistical Analysis

The data are expressed as the means ± Standard deviation (SD). Analyses of outcome measures were performed according to the per-protocol analysis set. Comparisons between the values for the placebo and yogurt group, and the changes from baseline were performed using an unpaired student’s *t*-test. Comparisons between the baseline and values after intake (2 W, 4 W, 6 W, 8 W, 10 W, 12 W) within each group were performed using a paired student’s *t*-test. The occurrence and duration of the common cold during the intake period was compared between the groups using Fisher’s exact test and Mann-Whitney U test. Correlation analyses between the NK cell activity and the VAS scores were performed by Peason’s rank test. Statistical analyses were performed using the statistical software package, Ekusel–Toukei 2015 (Social Survey Research Information Co., Ltd., Tokyo, Japan). Two-tailed tests were performed at a significance level of 5%.

## 3. Results

**Figure 2:**
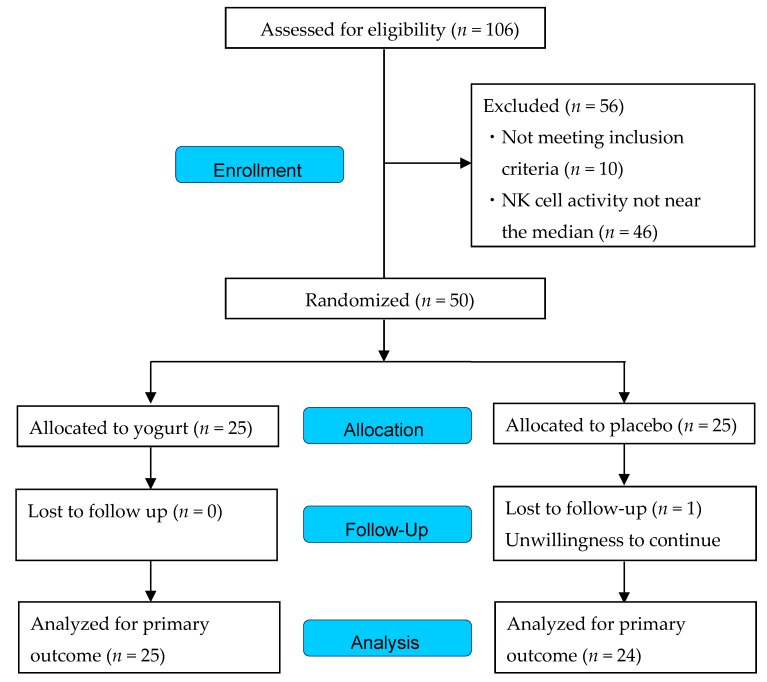
Study flow diagram.

In total, 106 males gave written informed consent and were assessed for eligibility. The participant flow through the study is shown in Figure 2. Fifty of the 106 males were enrolled and randomized to receive the yogurt (*n* = 25) or placebo (*n* = 25). After 4 weeks of intervention, 1 participant was lost to follow-up, but 49 completed the study and were included in the analysis.

The baseline characteristics of the study population are shown in Table 2.

No significant differences with respect to age, BMI, NK cell activity, and VAS scores associated with fatigue were observed between the two groups at the baseline.

No detectable side effects were observed in both the groups.

### 3.1. Questionnaires

**Figure 3:**
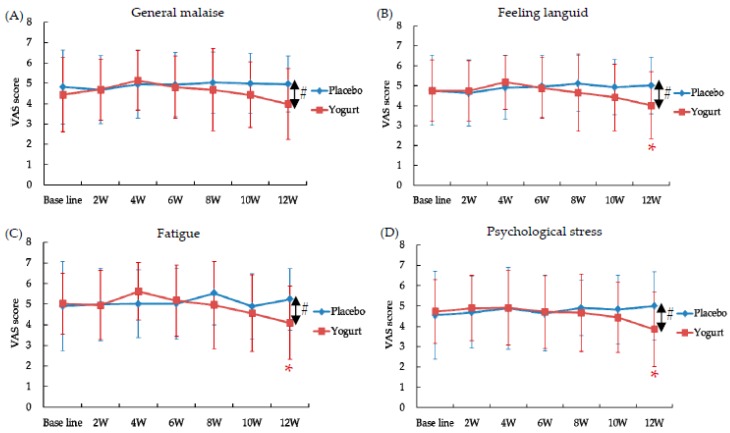
Effects of intake of yogurt fermented with *L. bulgaricus* OLL1073R-1 on the visual analogue scale (VAS) scores for general malaise (**A**), feeling languid (**B**), fatigue (**C**), and psychological stress (**D**). Data are presented as the mean ± standard deviation. * *p* < 0.05 compared to the baseline by paired *t*-test. # *p* < 0.05 between the groups by *t*-test.

The VAS scores for “general malaise”, “feeling languid”, “fatigue” and “psychological stress” after the 12-week intake period (12 W), were significantly lower in the yogurt group compared to the placebo group (Figure 3). The scores for “feeling languid”, “fatigue,” and “psychological stress” were decreased significantly from the baseline in the yogurt group (Figure 3B–D). No significant differences between the groups were observed in the other items in VAS scores, POMS, and Face scale. One subject from each group did not feel summer heat fatigue during the intake period.

### 3.2. NK Cell Activity

**Figure 4:**
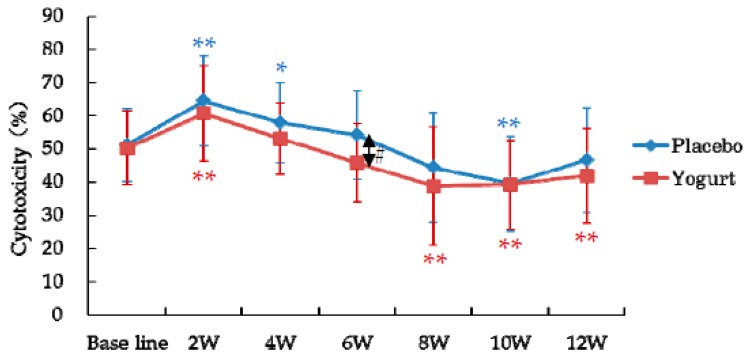
NK cell activity in the yogurt (*n* = 25) and placebo (*n* = 24) groups during the study. Data are presented as the mean ± standard deviation. * *p* < 0.05, ** *p* < 0.01 compared to the baseline by paired *t*-test; # *p* < 0.05 compared between the groups by *t*-test.

NK cell activity increased from the baseline in the first 2 weeks of intake (2 W) and then decreased in both the groups (Figure 4). In the yogurt group, after 6 weeks of intake, NK cell activity was lower than that in the placebo group (Figure 4). No correlations were detected at all points between the NK cell activity and the VAS scores for “general malaise,” “feeling languid,” “fatigue,” and “psychological stress”.

### 3.3. Catching Colds

Incidences, episodes, and the duration of the common cold in the yogurt group tended to be lower than those in the placebo group, but the differences were not significant (Table 3).

### 3.4. Autonomic Nervous System

**Figure 5:**
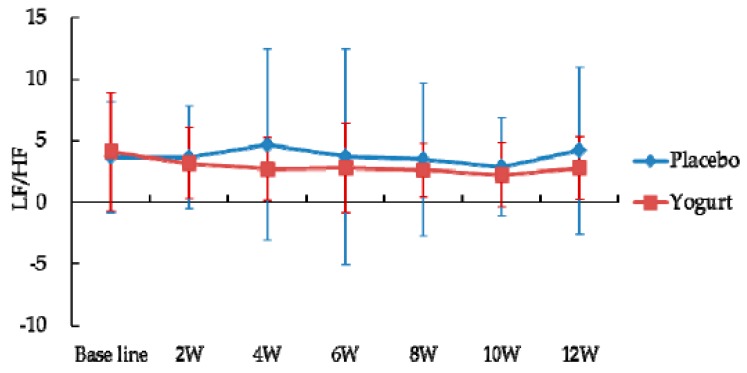
Effect of intake of yogurt fermented with *L. bulgaricus* OLL1073R-1 on autonomic nervous balance. Low frequency (LF) and high frequency (HF) indicate sympathetic and parasympathetic nervous, respectively. Data are presented as the mean ± standard deviation.

In the yogurt group, LF (Low frequency)/HF (High frequency) indicate the balance of the autonomic nervous system, which was better maintained (lower) compared to the placebo group during the intake period (Figure 5). However, the difference was not significant.

### 3.5. Blood Pressure

**Figure 6:**
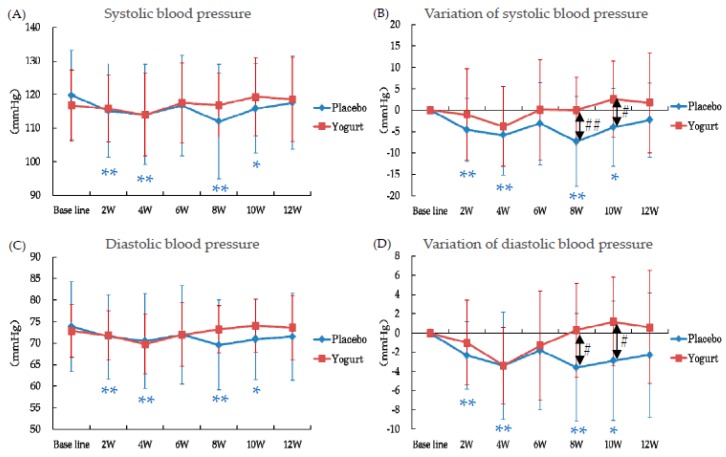
Effects of intake of yogurt fermented with *L. bulgaricus* OLL1073R-1 on systolic blood pressure (**A**), variation of systolic blood pressure (**B**), diastolic blood pressure (**C**), and variation of diastolic blood pressure (**D**). Data are mean ± standard deviation. * *p* < 0.05, ** *p* < 0.01 compared to the baseline by paired *t*-test. # *p* < 0.05 between the groups by *t*-test. ## *p* < 0.01 between the groups by *t*-test.

Blood pressures in the placebo group decreased significantly and were maintained at lower than baseline during the intake period (Figure 6). On the other hand, in the yogurt group, blood pressure decreased to 4 W but then increased and was recovered to the baseline level (Figure 6). At 8 W and 10 W, variation of blood pressures in the yogurt group was significantly higher than that in the placebo group (Figure 6B,D).

## 4. Discussion

In the present study, intake of yogurt fermented with *L. bulgaricus* OLL1073R-1 improved the typical symptoms of summer heat fatigue such as “general malaise” and “feeling languid” at the end of the intake period, compared with the placebo intake group. Moreover, our study suggests that yogurt intake probably affected the autonomic nervous system. The autonomic nervous system is known to regulate various physiological functions [19], and its imbalance has been reported in chronic fatigue syndrome [20]. In the present study, the LF/HF, which indicates autonomic nervous system balance, was found to be better maintained (lower) in the yogurt group and from 6 W to 12 W, when the temperatures fluctuated repeatedly, recovery of blood pressure was detected only in the yogurt group. Thus, yogurt may tune the balance of the autonomic nervous system and relieve the physical and mental disorders induced by seasonal changes. 

On the other hands, the NK cell activity was not increased by the yogurt consumption and not correlated with the VAS scores related to summer heat fatigue. In patients with chronic fatigue syndrome, decrease in the NK cell activity [2] and imbalance in autonomic nervous system [20] have been reported. In summer heat fatigue, fatigues and imbalance in autonomic nervous system are induced by the seasonal changes; however, these may not induce severe dysfunctions in the immune system and thus, differ from chronic fatigue syndrome. Changes in NK cell activity observed in this study were almost the same in the both groups and these may be the usual changes occurring during summer to early autumn.

Mechanisms of beneficial effects yogurt fermented with *L. bulgaricus* OLL1073R-1 for summer heat fatigue still remain unclear, but one of the active components may be EPS produced by *L. bulgaricus* OLL1073R-1. There are several reports that polysaccharides purified from mushrooms, seaweed, vegetables, Chinese herbs, etc. exert an anti-fatigue effect [9,10,11,12,13,21,22,23,24]. These reports showed effects on physical fatigue via prolonging exhaustion time in animal studies. It is well known that oxidative stress, energy source depletion, and excessive metabolite accumulation are involved in the occurrence of physical fatigue [25,26,27]. With the mushroom polysaccharide, antioxidant activity, free radical scavenging activity, immunomodulatory activity, and maintenance of normal liver function were involved in the anti-fatigue effect [28]. *L. bulgaricus* OLL1073R-1 EPS probably exhibits anti-fatigue effects via similar mechanisms. The reasons are that *L. bulgaricus* OLL1073R-1 EPS contain immunostimulatory phosphopolysaccharides [29] and that antioxidant activity and free radical scavenging activity have been shown in phosphopolysaccharides derived from *Lactococcus lactis* subsp. *lactis* [30]. Antioxidant activities have also been reported in EPS from another lactic acid bacterium [31,32,33,34].

This study suggests that yogurt fermented with *L. bulgaricus* OLL1073R-1 ameliorates summer heat fatigue and has the potential to affect the autonomic nervous system. These findings provide new insights into the health-promoting effects of yogurt fermented with *L. bulgaricus* OLL1073R-1. However, some limitations exist in this human trial. First, the participants are only men and a relatively small number. Second, because the immune markers used in this study is only the activity of NK cells, it is not clear whether summer heat fatigue is associated with the dysfunction of the immune system. Therefore, further investigations are required to validate our finding that daily intake of yogurt containing EPS reduces the fatigue felt in daily life, including summer heat fatigue.

## 5. Conclusions

Intake of yogurt fermented with *L. bulgaricus* OLL1073R-1 improved fatigue and the typical symptoms of summer heat fatigue such as “general malaise” and “feeling languid” in early autumn. These results suggest this yogurt ameliorates summer heat fatigue. However, further investigations are needed to determine the effect of the yogurt on summer heat fatigue and its mechanisms.

## Figures and Tables

**Table 1:** Composition of the 100 mL of test drinks.

Nutrients (g)	Yogurt	Placebo
Carbohydrate	12.4	11.7
Protein	3.2	4.7
Fat	0.6	0.6
Energy (kcal)	67.9	69.2

**Table 2:** Baseline characteristics of the study population.

Characteristics	Yogurt (*n* = 25)	Placebo (*n* = 24)	*p* Value
Age (years)	40.1 ± 6.0	39.8 ± 6.2	0.89
Body mass index (kg/m^2^)	23.5 ± 2.7	22.2 ± 2.7	0.09
NK cell activity (%)	50.3 ± 11.1	51.2 ± 10.9	0.78
VAS score for general malaise	4.4 ± 1.8	4.8 ± 1.8	0.47
VAS score for feeling languid	4.7 ± 1.6	4.8 ± 1.8	0.96
VAS score for fatigue	5.0 ± 1.5	4.9 ± 2.0	0.82

Data are presented as means ± standard deviation. Comparisons between groups were performed using Student’s *t*-test. NK, Natural killer; VAS, Visual analogue scale.

**Table 3:** Incidence and duration of catching colds in the participants.

	Yogurt (*n* = 25)	Placebo (*n* = 24)	*p* Value
Incidences	4	5	-
Incidence rate (%)	16.0	20.8	0.73
Episodes	5	7	-
Cumulative durations (days)	14	45	-
Duration per episode (days)	2.8 ± 1.9	6.4 ± 8.0	0.29

The duration per episode is presented as the mean ± standard deviation. Comparisons between groups were performed using Student’s *t*-test or Fisher’s exact test.
